# Nitrate Supply-Dependent Shifts in Communities of Root-Associated Bacteria in *Arabidopsis*

**DOI:** 10.1264/jsme2.ME17031

**Published:** 2017-11-30

**Authors:** Noriyuki Konishi, Takashi Okubo, Tomoyuki Yamaya, Toshihiko Hayakawa, Kiwamu Minamisawa

**Affiliations:** 1 Graduate School of Agricultural Science, Tohoku University 468–1 Aramaki Aza Aoba, Sendai, Miyagi 980–0845 Japan; 2 Division for Interdisciplinary Advanced Research and Education, Tohoku University 6–3 Aramaki Aza Aoba, Aoba-ku, Sendai, Miyagi 980–8578 Japan; 3 Institute for Agro-Environmental Sciences, National Agriculture and Food Research Organization 3–1–3 Kannondai, Tsukuba, Ibaraki 305–8604 Japan; 4 Graduate School of Life Sciences, Tohoku University Katahira, 2–1–1 Aoba-ku, Sendai, Miyagi 980–8577 Japan

**Keywords:** root-associated bacterial communities, *Arabidopsis*, nitrate, NIN-like protein, TCP20

## Abstract

Root-associated bacterial communities are necessary for healthy plant growth. Nitrate is a signal molecule as well as a major nitrogen source for plant growth. In this study, nitrate-dependent alterations in root-associated bacterial communities and the relationship between nitrate signaling and root-associated bacteria in *Arabidopsis* were examined. The bacterial community was analyzed by a ribosomal RNA intergenic spacer analysis (RISA) and 16S rRNA amplicon sequencing. The *Arabidopsis* root-associated bacterial community shifted depending on the nitrate amount and timing of nitrate application. The relative abundance of operational taxonomic units of 25.8% was significantly changed by the amount of nitrate supplied. Moreover, at the family level, the relative abundance of several major root-associated bacteria including *Burkholderiaceae*, *Paenibacillaceae*, *Bradyrhizobiaceae*, and *Rhizobiaceae* markedly fluctuated with the application of nitrate. These results suggest that the application of nitrate strongly affects root-associated bacterial ecosystems in *Arabidopsis*. Bulk soil bacterial communities were also affected by the application of nitrate; however, these changes were markedly different from those in root-associated bacteria. These results also suggest that nitrate-dependent alterations in root-associated bacterial communities are mainly affected by plant-derived factors in *Arabidopsis*. T-DNA insertion plant lines of the genes for two transcription factors involved in nitrate signaling in *Arabidopsis* roots, *NLP7* and *TCP20*, showed similar nitrate-dependent shifts in root-associated bacterial communities from the wild-type, whereas minor differences were observed in root-associated bacteria. Thus, these results indicate that NLP7 and TCP20 are not major regulators of nitrate-dependent bacterial communities in *Arabidopsis* roots.

Root-associated microbes, including endophytes and epiphytes, are crucial for healthy plant growth because they promote nutrient acquisition and stress tolerance (5, 8, 50, 58). Thus, plant-associated microbes contribute to sustainable agriculture (57). In modern agriculture, nitrogen fertilizers are generally needed in order to attain high crop yields; however, previous studies demonstrated that this form of fertilization often changes microbial communities associated with plants, including soybean (23, 24), rice (14, 51, 56), maize (18, 43), wheat (42), and sugarcane (60). Nitrate, which is the dominant nitrogen form in aerobic soil, is a major nitrogen source for plant growth, but also functions as a signal molecule in plants (2, 45). Thus, nitrate is regarded as a key compound that affects plant-microbe relationships (23).

Nitrate-dependent symbiotic relationships between legumes and rhizobia are regulated by the nodule inception (NIN) transcription factor (54, 55), several glycopeptides (37, 39), and certain phytohormones (9, 16). However, limited information is currently available on the factors regulating nitrate-dependent relationships among root-associated bacteria and non-legume plants.

The molecular mechanisms underlying plant nitrate signaling have been intensively examined over the last decade using *Arabidopsis thaliana* as a model. Nitrate signaling is mediated by the transcription factors ANR1 (17, 41, 61), LBD37/38/39 (44), NLP6/7 (28, 34), SPL9 (30), TGA1/4 (1), and TCP20 (19), by the nitrate transporter NRT1.1 (59), by the kinases CIPK8 and CIPK23 (21, 22), by cytokinins (27, 49), and by glycopeptides (3). NLP6 and 7 are homologous genes to NIN in the model legume *Lotus japonicus* (52). Nine genes of NIN-like proteins (NLPs) are coded in the *Arabidopsis* genome (52). NLP7 has the ability to bind to a nitrate-responsive *cis*-element (28), and regulates the expression of more than 90% of the primary nitrate-responsive genes in *Arabidopsis* roots after the application of nitrate (34). Thus, NLP7 is a master regulator of nitrate-responsive genes in *Arabidopsis* roots (34). TEOSINTE BRANCHED1/CYCLOIDEA/PROLIFERATING CELL FACTOR1–20 (TCP20) is a regulator of nitrate-dependent changes in the root system architecture (19). In order to adapt to the heterogeneous nitrate conditions in natural soils, *Arabidopsis* elongates the lateral roots in high-nitrate patches, while suppressing elongation in low-nitrate patches (41). This nitrate-dependent systemic modification of lateral roots disappeared in *tcp20* mutants (19). Comparisons between *tcp20* mutants and *nlp7* mutants, which are defective in the local control of root growth, but not in the systemic root growth response for nitrate, indicated that TCP20 functions independently of NLP7 (19).

Previous studies investigated root-associated bacterial communities in *Arabidopsis* (6, 7, 32, 33, 53) and revealed that the community structures of root-associated bacteria are related to soil properties, plant ecotypes (7, 33), and salicylic acid signaling (32). However, the effects of nitrate on *Arabidopsis* root-associated bacteria have not yet been clarified. In the present study, we examined whether plant genes for nitrate signaling are involved in nitrate-dependent community shifts in root-associated bacteria in *Arabidopsis*. In order to achieve this, root-associated bacterial communities were investigated under various nitrate levels via a ribosomal RNA intergenic spacer analysis (RISA). The bacterial community was determined via 16S rRNA amplicon sequencing at the family level. The relative abundance and potential function of root-associated bacteria were then compared between wild-type *Arabidopsis* and T-DNA insertion lines of the *NLP7* and *TCP20* genes under low- and high-nitrate conditions.

## Materials and Methods

### Soil collection and preparation

The top 10 cm of a gray lowland soil was collected with a shovel from an experimental field in Kashimadai, Miyagi, Japan (38°27′39.37″N, 141°5′33.33″E; altitude 4 m) and transported to the laboratory in plastic containers at an ambient temperature (24). The soil was sampled in July 2013 and then air-dried in a greenhouse for 15 d. Visible weeds, twigs, worms, insects, and other organisms were removed from air-dried soil, which was then crushed with a rubber mallet to a fine consistency. The soil was then stored at room temperature in a closed polyethylene bucket until used.

### Growth and harvest

**Fig. 1:**
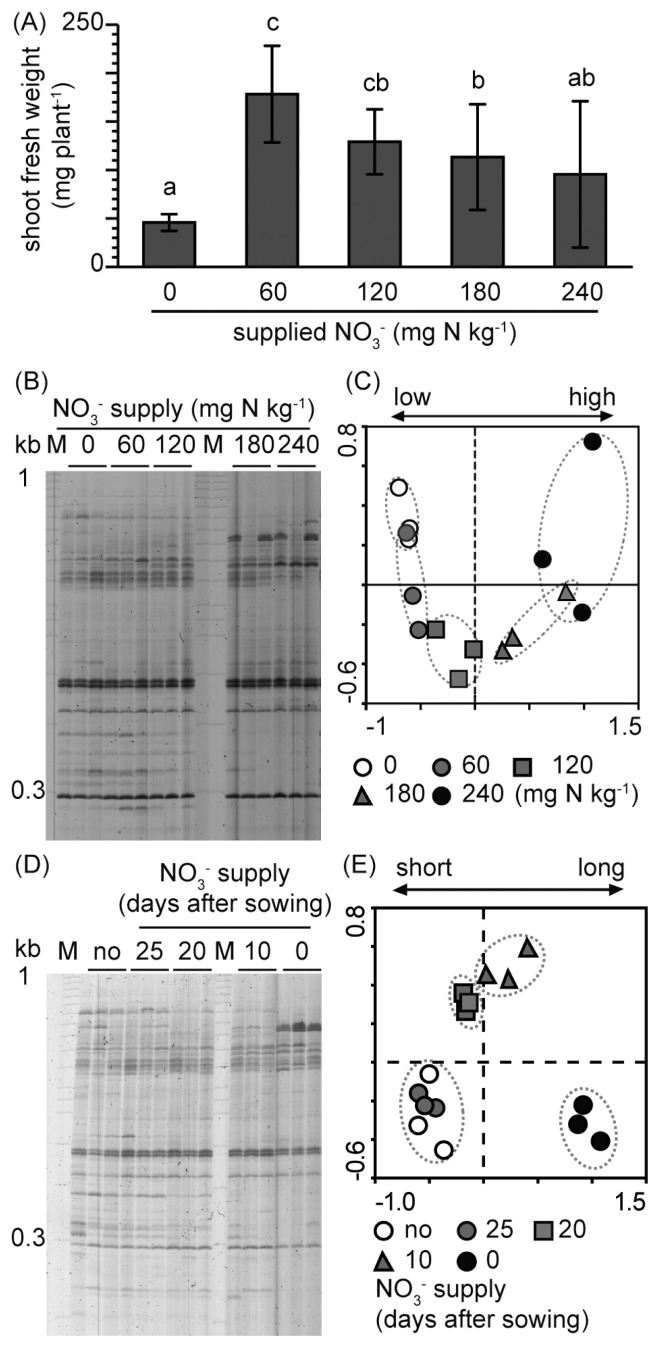
RISA profiles of nitrate-dependent shifts in *Arabidopsis* root-associated bacterial communities. (A) Nitrate amount-dependent shoot growth. Data are calculated as means±standard deviation (*n*=15). (B) RISA profiles of nitrate amount-dependent shifts in root-associated bacterial communities. (C) PCA of data in (B); PC1 explained 38.5% of the variability and PC2 explained 10.8%. (D) RISA profiles of nitrate application timing-dependent shifts in root-associated bacterial communities. Nitrate (240 mg N kg^−1^) was added only once, either before sowing or 15, 20, or 25 d after sowing. (E) PCA of data in (D); PC1 explained 31.1% of the variability and PC2 explained 12.1%. Plants were grown under various nitrate conditions for 30 d.

*Arabidopsis* ecotype Col-0 was used as the wild-type plant for every analysis. Seeds were sterilized in 70% ethanol before sowing (29). Plants were cultivated in sterilized 10×9×8 cm plastic containers (Steri Vent high container; Duchefa Biochemie B.V., Amsterdam, the Netherlands), each containing 250 g of air-dried soil. Five to eight seeds were sown in 5 respective points in each container (four corners and the center) and 100 mL of pure water was then added. When nitrate was supplied, various (0, 60, 120, 180, and 240 mg N kg^−1^ soil) amounts of sterilized 1 M KNO_3_ solution were added to the container. Nitrate was only added once: before sowing or 15, 20, or 25 d after sowing. As shown in Fig. 1B and C, the no nitrate added condition was defined as a low-nitrate (LN) condition, while the 240 mg N kg^−1^ nitrate applied condition was defined as a high-nitrate (HN) condition. Five to 7 d after sowing, the 5 healthiest plants in each container were selected, and the others were removed. A plastic cover was placed loosely on each container for the first 5 d after sowing to prevent drying, and it was then replaced with a paper cover. Pure water (30–50 mL) was supplied 10, 15, 20, and 25 d after sowing. Plants were grown in a culture room (14 h/10 h light/dark cycle, 19–22°C, and 160 μmol m^−2^ s^−1^ photosynthetic photon flux density). Containers were rotated on the shelf every 5 d. Plants other than *Arabidopsis* growing in the containers were removed at these times.

Plants were harvested 30 d after sowing for every analysis. At the same time, bulk soil (400–500 mg) was collected from places in the container without plants (5–10 mm depth from the soil surface). Immediately after cutting the hypocotyl, the shoot fresh weight was measured with an electronic balance (CPA324; Sartorius Co., Göttingen, Germany). The container was then inverted and all soil was placed on a plastic plate in order to allow for the roots to be removed with tweezers. Roots in each container (*i.e.*, those of the five plants) were placed in a clean 50-mL tube containing 25 mL phosphate buffer (per L: 5.4 g of NaH_2_PO_4_, 8.8 g of Na_2_HPO_4_, and 200 μL of Silwet L-77). Tubes were then vortexed at the maximum speed for 15 s, which released most of the rhizosphere soil from the roots. Root samples included epiphytes and endophytes (33). Water was removed from the roots using a paper towel, the dried roots were placed in a sterile 2-mL tube with zirconia beads (diameter of 5 mm) and then immediately frozen at −80°C until used.

### Evaluation of soil chemical properties

Soil pH, total nitrogen, hot water-extracted nitrogen, nitrate, and ammonium contents were assessed by the Tokachi Nokyoren Agricultural Research Institute (Obihiro, Hokkaido, Japan). In the present study, the value of hot water-extracted nitrogen was used as an approximation of available nitrogen.

### DNA extraction

DNA was extracted from root and soil samples using a FastDNA Spin Kit for Soil (MP Biomedicals LLC, Santa Ana, CA, USA) according to the manufacturer’s instructions. Root samples were frozen in liquid nitrogen then milled with Tissue Lyser II (Qiagen K. K., Hilden, Germany) at 23 Hz for 1.5 min. DNA was quantified using a spectrophotometer (NanoDrop 2000; Thermo Fisher Scientific K.K., Waltham, MA, USA), then stored at −80°C until the PCR analysis.

### Ribosomal RNA intergenic spacer analysis

RISA was performed as described by Saito *et al.* (48) using two primer sets: the bacterial primer for ITSF/ITSReub and the fungal primer for ITS1F/ITS4. ITSF and ITS1F were labeled by 6-carboxyfluorescein-aminohexyl amidite. After electrophoresis, digital fingerprinting images were obtained with a fluorescent scanner (FLA-2000; Fujifilm, Tokyo, Japan). Band patterns were analyzed using FPQuest Software (Bio-Rad, Hercules, CA, USA). A principal-component analysis (PCA) was performed using CANOCO (version 4.5 for Windows; Microcomputer Power, Ithaca, NY, USA) with default parameters (except that intersample scaling was used) to generate ordination plots based on the scores of the first two principal components.

### RT-PCR

Plants were grown on agarose plates with half-strength Murashige and Skoog medium agar for 25 d. Total RNA was extracted from whole plants using Sepasol-RNA I Super G (Nacalai Tesque, Kyoto, Japan). Reverse transcription and a DNase treatment were performed using a PrimeScript RT Reagent Kit with genomic DNA Eraser (Takara Bio, Otsu, Japan) with 470 ng of total RNA in a 10 μL final volume, according to the manufacturer’s instructions. Twice-diluted reverse transcription solution was used for the PCR template. Ex *Taq* (Takara Bio) was used for PCR. *TCP20* (19) and *UBQ2* (29) were amplified using the above primers and *NLP7* using NLP7_RTPCR_F (5′-AGCGTGGGAAGACTGAGAAA-3′) and NLP7_RTPCR_R (5′-TTGGGGGAGCGTATAAGTTG-3′).

### 16S rRNA amplicon sequencing

16S rRNA amplicon sequencing was performed as described previously (11). The V4 region of the bacterial and archaeal 16S rRNA gene was amplified using a two-step PCR procedure. The following primers were used in the first step: 515F (5′-ACACTCTT TCCCTACACGACGCTCTTCCGATCTGTGCCAGCMGCCGC GGTAA-3′) and 806R (5′-GTGACTGGAGTTCAGACGTGTGC TCTTCCGATCT-GGACTACHVGGGTWTCTAAT-3′), and eight forward and four reverse primers in the second step: flow cell binding sites (forward; AATGATACGGCGACCACCGAGATCTACAC, reverse; CAAGCAGAAGACGGCATACGAGAT), Illumina indexes (forward; D501–D508, reverse; D703–D706), and the sequencing primer-binding site (forward; ACACTCTTTCCCTACACGACGC, reverse; GTGACTGGAGTTCAGACGTGTG). Ex *Taq* HS (Takara Bio) was used for first and second PCR. First PCR was performed as follows: initial denaturation at 94°C for 2 min followed by 24 cycles of 94°C at 30 s, 51°C for 30 s, and 72°C for 30 s, with final extension at 72°C for 5 min. The amplification products were purified using an AMPure XP bead (Beckman Coulter, Brea, USA). Second PCR was performed using purified DNA as a template as follows: initial denaturation at 94°C for 2 min followed by 10 cycles of 94°C at 30 s, 60°C for 30 s, and 72°C for 30 s, with final extension at 72°C for 5 min. The amplified products were used for sequencing after purification using AMPure XP beads (Beckman Coulter). PCR and amplicon sequencing were performed by FASMAC Co. (Kanagawa, Japan) using the Illumina MiSeq platform, following the 2×250 bp paired-end sequencing protocol (Illumina, San Diego, CA, USA). 16S rRNA gene sequences were processed using MacQIIME 1.9.1 (10), and paired-end sequences assembled using the pick_de_novo_otus command. Low-quality reads were filtered using the multiple_split_libraries_fastq command. Chimeric sequences were removed using USEARCH6.1 software (15). The remaining sequences were clustered into operational taxonomic units (OTUs) at 97% similarity using the pick_open_reference_otus command in the Greengenes reference database version 13.5. Contaminant sequences from the host plant, classified as chloroplasts or mitochondria, were removed. In order to normalize the number of sequences per sample, the random subsampling of 4,724 sequences from each sample was applied for further analyses. Three independent samples were used per genotype and condition. The number of sequences in each sample after filtering is shown in Table S1.

A principal-coordinates analysis (PCoA) was performed on weighted UniFrac distance matrixes. The rarefaction procedure was repeated 100 times to compute the number of OTUs, Shannon’s diversity index, and Simpson’s index. Functional profiles of the bacterial community were predicted using the PICRUSt program (31) according to the protocol provided online (http://picrust.github.io/picrust/tutorials/otu_picking.html). Predicted gene functions were summarized based on the KEGG Pathway database (http://www.genome.jp/kegg/pathway.html). Statistical comparisons were performed using Welch’s *t*-test with *P*<0.05 considered to be significant.

### Accession numbers

Raw sequence data were deposited in the NCBI Sequence Read Archive under accession numbers SRR5182883–SRR5182906.

## Results

### Nitrate altered root-associated bacterial communities in

Arabidopsis Plants were treated with different amounts of nitrate (0, 60, 120, 180, or 240 mg N kg dry soil^−1^), which corresponded to 6 to 24 kg of nitrogen fertilizer in a 10 a field (38). A low amount of nitrate (60 or 120 mg N kg^−1^) promoted better shoot growth than high-nitrate (180 and 240 mg N kg^−1^) conditions (Fig. 1A). RISA and subsequent PCA showed no significant differences in root-associated bacterial communities between the 0 and 60 mg N kg^−1^ treatments (Fig. 1B, and C), whereas community differences were observed between the 120, 180, and 240 mg N kg^−1^ treatments and the treatment that received no nitrate. The community shift became more prominent as the nitrate amount increased. Since the most notable difference in RISA profiles was observed between the 0 and 240 mg N kg^−1^ treatments, these conditions were defined as low-(LN) and high-nitrate (HN) conditions, respectively. (Note that the nitrate concentration of LN soil was 3.1 mg N kg^−1^ after planting [Table 1].)

We then examined the effects of nitrate supply timing on root-associated bacterial communities. The communities were not affected 5 d after the nitrate treatment, whereas distinct communities were found 10 d or more after the nitrate treatment (Fig. 1D and E). Community shifts were more prominent with increases in time after the application of nitrate than after the no nitrate treatment (Fig. 1E).

Soil pH and total nitrogen, available nitrogen, nitrate, and ammonium contents were also assessed, and revealed significant increases in total nitrogen and nitrate contents in HN soil (*P*<0.05). In contrast, lower nitrate and significantly higher ammonium contents were observed in LN soil (*P*<0.05) than in unplanted soil (Table 1). The pH of HN soil was significantly less (*P*<0.05), whereas that of LN soil was significantly higher (*P*<0.05) than that of unplanted soil. No significant differences were observed in the amounts of available nitrogen between the soils.

### Nitrate-responsive bacteria in *Arabidopsis* roots

**Fig. 2:**
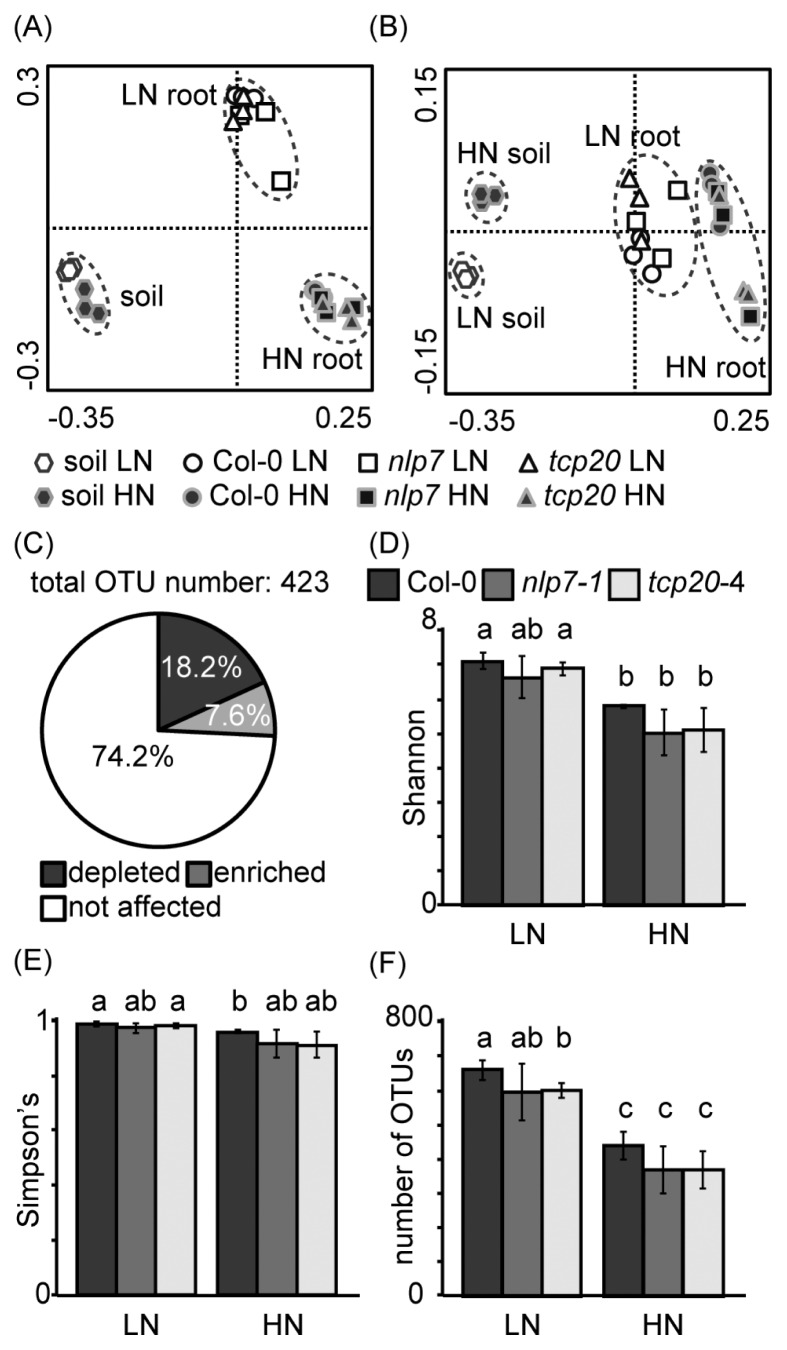
PCoA plots for 16S rRNA genes of bacteria associated with wild-type *Arabidopsis*, *nlp7-1*, and *tcp20-4* roots and those in bulk soil. (A) PC1 vs. PC2 plot; PC1 explained 45.2% of the variability and PC2 explained 38.0%. (B) PC1 vs. PC3 plot; PC3 explained 6.8% of the variability. Low-nitrate (LN) conditions were indicated by open symbols and high-nitrate (HN) conditions were indicated by closed symbols. The ordination was constructed using UniFrac distances. (C) The percentage of nitrate-affected OTUs in wild-type (Col-0) roots. The relative abundance of OTUs in wild-type roots of the LN treatment was compared with that of HN. (D) Shannon’s diversity index, (E) Simpson’s diversity index, and (F) the number of OTUs; different letters indicate significant differences (*P*<0.05) between bars, according to Welch’s *t*-test. Data represent means±standard deviation (*n*=3). LN, no nitrate application; HN, high (240 mg N kg^−1^) nitrate application.

In order to identify the bacterial groups for which abundance was affected by the nitrate supply, root-associated bacterial communities were analyzed using 16S rRNA amplicon sequencing under LN and HN conditions. Bacterial communities in bulk soil samples were also examined in order to clarify the effects of nitrate on free-living bacterial communities. According to PCoA plots, root-associated bacterial communities were different from those in bulk soil, as explained by PC1 (Fig. 2A). Nitrate-dependent shifts in root-associated bacterial communities were also observed by 16S rRNA amplicon sequencing (Fig. 2A and B). Bacterial communities in bulk soil appeared to differ between the LN and HN conditions; however, the community shift was smaller than that of root-associated bacteria. The bacterial community shift in bulk soil was observed along the PC3 axis, whereas that of roots was observed along the PC2 axis.

Amplicon sequencing produced 423 OTUs, each of which contained at least five reads in the root samples. The relative abundance of OTUs of 25.8% was changed by the application of nitrate; 18.2% of the OTUs were decreased by nitrate, whereas 7.6% were increased (*P*<0.05, Fig. 2C). The indexes of alpha diversity (Shannon’s, Simpson’s, and number of OTUs) in wild-type roots were decreased by the application of nitrate (*P*<0.05, Fig. 2D–F). *Proteobacteria*, *Bacteroidetes*, and *Firmicutes* were the major phyla of root-associated bacteria for which relative abundance was altered (Table 2) and all are major phyla in *Arabidopsis* roots (6, 7, 33). The most abundant families (more than 5% of the average relative abundance) are shown in Table 3. The relative abundance of *Burkholderiaceae* and *Paenibacillaceae* increased by more than 100-fold with the application of nitrate, whereas that of *Comamonadaceae*, *Caulobacteraceae*, *Sphingomonadaceae*, *Bradyrhizobiaceae*, *Rhizobiaceae*, *Chitinophagaceae*, and *Cytophagaceae* decreased (*P*<0.05).

### Effects of *NLP7* and *TCP20* genes on root-associated bacterial communities

Four independent T-DNA insertion lines for *NLP7* (*nlp7-1* and *nlp7-2*) and *TCP20* (*tcp20-2* and *tcp20-4*) were obtained from the Arabidopsis Biological Resource Center using Col-0 as the genetic background. Previously, *nlp7-1* and *nlp7-2* were identified as knockout and knockdown mutants, respectively (12), and *tcp20-2* and *tcp20-4* as knockout mutants (19). An RT-PCR analysis showed that *NLP7* or *TCP20* gene expression disappeared due to the respective T-DNA insertion (Fig. S1). Moreover, the expression levels of *NLP7* were so low in *nlp7-2* that they were not detectable by our RT-PCR.

**Fig. 3:**
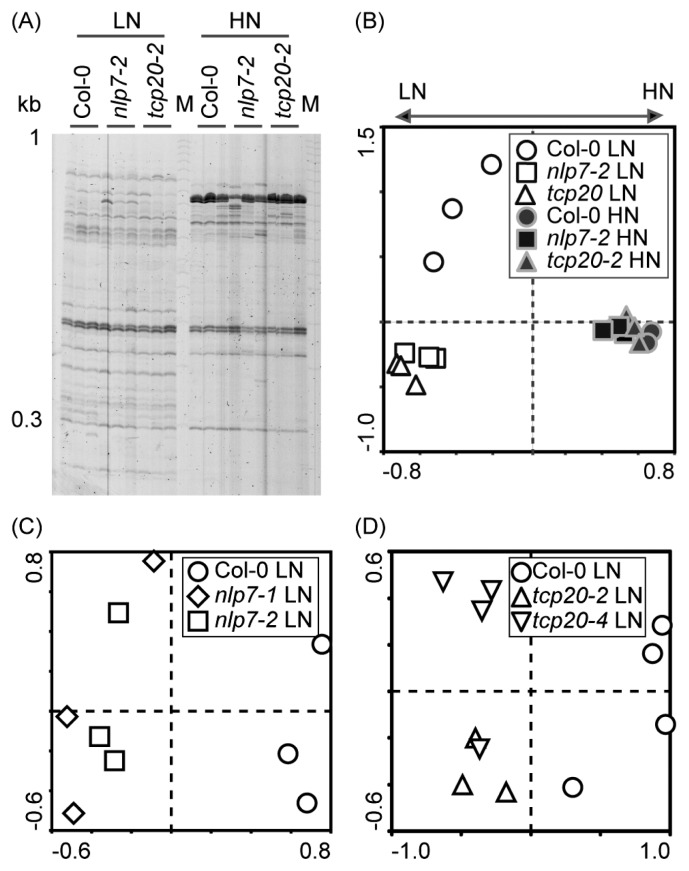
RISA profiles of root-associated bacterial communities in *Arabidopsis* wild-type (Col-0) and *nlp7* and *tcp20* mutants. (A) RISA profiles of root-associated bacterial communities in the wild-type, *nlp7-2*, and *tcp20-2* under low-nitrate (LN) and high-nitrate (HN) conditions. (B) PCA of the data in (A); PC1 explained 31.7% of the variability and PC2 explained 17.7%. (C) PCA of the RISA bacterial profile of the wild-type, *nlp7-1*, and *nlp7-2* under LN conditions; PC1 explained 24.2% of the variability and PC2 explained 17.0%. (D) PCA of RISA bacterial profile of the wild-type, *tcp20-2*, and *tcp20-4* under LN conditions; PC1 explained 33.3% of the variability and PC2 explained 10.8%. LN: No nitrate application, HN: Nitrate (240 mg N kg^−1^) application.

The community structures of root-associated bacteria were compared between wild-type and insertion lines (*nlp7-2* and *tcp20-2*) using RISA (Fig. 3A). In PCA plots, the bacterial communities of insertion lines differed from those of the wild-type under LN conditions, but not under HN conditions (Fig. 3B). Similar differences were observed in the second insertion lines for the *NLP7* (Fig. 3C) and *TCP20* (Fig. 3D) genes under LN conditions. Fungus-specific RISA profiles showed only one band in all genotypes, and the fungus signal was not affected by nitrate application (Fig. S2).

In order to identify the bacterial groups for which abundance was affected by the knockout of the *NLP7* or *TCP20* gene, bacterial communities were analyzed by 16S rRNA amplicon sequencing in the roots of the wild-type and two insertion lines (*nlp7-1* and *tcp20-4*). The PCoA plots did not show significant differences in bacterial communities between the wild-type and two insertion lines under LN or HN conditions (Fig. 2A and B). The number of OTUs in *tcp20-4* was lower in LN than in the wild-type (*P*<0.05, Fig. 2F), whereas no significant differences were observed in Shannon’s and Simpson’s indexes (Fig. 2D and E). In *nlp7-1*, these indexes were similar to those of the wild-type (Fig. 2D–F). Although PCoA plots did not show clear community differences between the wild-type and two insertion lines by 16S rRNA sequencing, a phylogenetic analysis showed significant differences in several minor taxonomic groups. The lack of NLP7 and TCP20 significantly affected the relative abundance of 4 classes, 13 families, 20 genera, and 48 OTUs (Table 2, 4, S2, and S3). Under LN conditions, the abundance of *Sphinogomonadaceae*, *Actinomycetales*, and *Norcardiodaceae* increased in *tcp20-4*, while that of *Bacteriovoracaceae* decreased in *nip7-1* (Table 4). In contrast, under HN conditions, the abundance of *Alphaproteobacteria*, including *Sphingomonadaceae*, decreased in *nlp7-1*, while that of *Chitinophagaceae*, *Nocardioidaceae*, and *Micrococcaceae* decreased in *tcp20-4* (Table 2 and 4). These results suggest that NLP7 and TCP20 affect the interaction with certain minor root-associated bacteria in *Arabidopsis*.

### Effects of NLP7 and TCP20 on functional genes in root-associated bacterial communities

In order to examine the knockout effects of the *NLP7* or *TCP20* gene on bacterial functions, functional gene frequencies were estimated from 16S rRNA sequencing and the gene contents of known bacterial genomes using PICRUSt (31). These genes were classified by functional units in the KEGG pathway for each nitrate condition. Under LN conditions, *tcp20-4* showed more than 40 differences in metabolic pathways from the wild-type, whereas *nlp7-1* showed only several differences (Table S4). The lack of TCP20 under LN conditions affected the pathways of secondary metabolism, lipid metabolism, xenobiotic biodegradation and metabolism, cellular processes, environmental information processing, and others (Table S4). Under HN conditions, the lack of NLP7 affected many pathways, including secondary metabolism, amino acid metabolism, carbohydrate metabolism, lipid metabolism, xenobiotic biodegradation and metabolism, and environmental information processing, whereas the lack of TCP20 showed only a few minor differences (Table 5). These results indicate that the knockout of the *NLP7* or *TCP20* gene affects some functions of the root-associated bacterial community.

## Discussion

The present study is the first to examine nitrate-dependent shifts in bacterial communities associated with *Arabidopsis* roots. Robinson *et al.* (42) proposed that fertilizer-dependent alterations in root-associated bacteria were regulated by two processes. Fertilizers may directly alter soil bacterial communities and, hence, affect the available pool from which bacteria colonize the plant. Furthermore, fertilizers may alter plant traits, such as growth and exudate production, thereby affecting the recruitment of endophytic communities. In the present study, root-associated bacterial communities were clearly different from those of bulk soil, and presented more significant community shifts 30 d after nitrate application (Fig. 2A and B). These results suggest that nitrate-dependent alterations in root-associated bacteria are mainly affected by plant-derived factors at least in *Arabidopsis* roots.

In the present study, the relative abundance of *Burkholderiaceae* and *Paenibacillaceae* increased by more than 100-fold with nitrate application (Table 3). Several members of *Burkholderiaceae* (26) and *Paenibacillaceae* (46, 47) have been suggested to compose the major groups of plant growth-promoting bacteria. For example, in rice roots, *Burkholderia kururiensis* KP23T contributes to nitrogen acquisition via nitrogen fixing functions in low nitrogen environments (26). However, the physiological implications of the interaction with *Burkholderiaceae* may differ between *Arabidopsis* and rice because the abundance of *Burkholderiaceae* decreases with the application of nitrogen in rice roots (26). Since a closed container was used in our cultivation system, soil was expected to be anaerobic, at least immediately after water supply. Under anaerobic conditions, *Burkholderiaceae* bacteria can reduce the nitrate (60). Since nitrate reduction is a highly energy-demanding process during nitrogen assimilation in plants (20), *Burkholderiaceae* may contribute to nitrate utilization in *Arabidopsis* roots through nitrate reduction. Members of *Paenibacillaceae* reportedly function as biocontrol agents for phytopathogens (46, 47) and also reduce biotic stress under high nitrate conditions (46, 47). Therefore, further analyses of the physiological effects of these nitrate-induced bacteria on *Arabidopsis* growth under high nitrate conditions are required.

Previous studies investigated nitrogen-dependent alterations in relationships with root-associated bacteria using high-resolution analyses, such as 16S rRNA amplicon sequencing and metagenome analyses (26, 42, 60). Reductions in *Alphaproteobacteria* by certain environmental factors are commonly observed in the roots of rice, wheat, sugarcane, and *Arabidopsis* (Table 2 and 3) (26, 42, 60), although the cultivation conditions and nitrogen forms supplied differed. *Bradyrhizobiaceae* and *Rhizobiaceae* include several nitrogen-fixing bacteria. The abundance of nitrogen-fixing *Alphaproteobacteria* was also found to decrease in the roots of rice (26) and legumes (40) with the application of nitrogen. Therefore, the mechanisms underlying nitrogen-dependent reductions in *Alphaproteobacteria* in roots appear to be conserved among higher plants.

In rice roots, the *Calcium/Calmodulin-Dependent Protein Kinase* (*CCaMK*) gene regulates the abundance of root-associated *Alphaproteobacteria* (25) and interactions with methanotrophs and nitrogen-fixing bacteria (4, 36). *CCaMK* is also an essential gene in the common symbiotic signaling pathway (CSP), which is necessary for developing a symbiotic relationship with rhizobia and mycorrhizal fungi in legumes (36). CSP is conserved in grasses, but not in *Arabidopsis* (62). We observed nitrate-dependent reductions in the relative abundance of *Alphaproteobacteria* in *Arabidopsis* roots (Table 2), suggesting that the interaction with *Alphaproteobacteria* is partially altered by a CSP-independent pathway. The CSP-independent regulation of endophytic *Alphaproteobacteria* has also been reported in rice (13).

*NLP* (52) and *TCP* (35) homologous genes are well-conserved transcription factors in higher plants, working independently of CSP. In the present study, the knockout of these genes significantly affected the abundance of several minor bacteria as well as a number of bacterial functions (Table 2, 4, 5, S2, S3, and S4). More than 80 functional pathways including amino acid, carbohydrate, lipid, and secondary metabolism were changed in the roots of the *nlp7* mutant under nitrate application (Table 5). In contrast, *tcp20* mutants showed several minor differences under LN conditions including alterations in root-associated bacterial communities (Fig. 3), bacterial relative abundance (Table 2, 4, S2, and S3), and functional gene frequencies (Table S4). However, these knockouts did not affect nitrate-dependent community shifts in root-associated bacteria (Fig. 2 and 3), suggesting that NLP7 and TCP20 are not the major regulators of nitrate-dependent alterations in root-associated bacteria.

The present study aimed to demonstrate the relationship between plant genes for nitrate signaling and nitrate-dependent community shifts in root-associated bacteria. However, this relationship remains unclear because the knockout of NLP7 and TCP20 did not exert strong effects on nitrate-dependent community shifts (Fig. 2). Nevertheless, nitrate-dependent shifts in bacterial communities in the roots of *Arabidopsis* strongly suggest the existence of plant regulator(s) of root-associated bacteria, which warrants further study.

## Supplementary materials



## Figures and Tables

**Table 1:** Characteristics of soil used in the present study

	soil pH	total nitrogen (%)	available nitrogen (mg kg^−1^)	nitrate (mg kg^−1^)	ammonium (mg kg^−1^)
Not planted	5.73±0.06^b^	0.12±0.00^a^	29.3±1.0^a^	5.9±0.3^b^	10.5±1.2^a^
At harvest					
LN	5.97±0.06^a^	0.12±0.01^ab^	28.0±3.2^a^	3.1±0.6^a^	15.7±0.8^b^
HN	5.17±0.06^c^	0.14±0.01^b^	30.6±0.7^a^	210.1±5.7^c^	11.9±2.4^ab^

LN, no nitrate application; HN, high (240 mg N kg^−1^) nitrate application. Values represent means±standard deviation (*n*=3). Within each column, different letters indicate significant differences at *P*<0.05 according to Welch’s *t*-test.

**Table 2:** The relative abundance of 16S rRNA genes of bacteria associated with roots of wild-type *Arabidopsis* and *nlp7-1* and *tcp20-4* mutants at the phylum, class, and order levels.

Taxon	LN			HN		
	
	Col-0	*nlp7-1*	*tcp20-4*	Col-0	*nlp7-1*	*tcp20-4*
*Proteobacteria*	54.5	57.6	51.0	59.8	69.3	72.8
*Alphaproteobacteria*#	6.2	6.3	6.5	3.0	2.0*	2.4
*Rhizobiales*#	3.7	3.4	3.4	0.9	0.7	0.9
*Caulobacterales*#	1.5	1.7	1.6	0.8	0.6	0.7
*Sphingomonadales*#	0.7	0.9	1.2*	1.0	0.6*	0.7
*Betaproteobacteria*#	36.6	43.0	33.3	49.7	63.3	64.9
*Burkholderiales*#	26.2	34.8	25.9	48.9	62.8	64.6
*Rhodocyclales*	9.5	7.4	6.7	0.2	0.0	0.0
*Deltaproteobacteria*#	7.3	5.5	6.9	0.5	0.3	0.3
*Myxococcales*#	5.6	4.2	5.5	0.2	0.1	0.1
*Gammaproteobacteria*	4.4	2.8	4.2	6.6	3.8	5.2
*Legionellales*	2.2	1.0	2.0	0.6	0.3	0.4
*Xanthomonadales*	2.0	1.6	2.0	5.8	3.3	4.6

*Actinobacteria*	6.8	13.3	17.0	10.9	7.5	6.1
*Actinobacteria*	6.6	13.0	16.7	10.7	7.4	5.9

*Bacteroidetes*#	13.8	12.7	13.4	4.0	3.1	2.0
*Saprospirae*#	7.5	7.4	8.3	3.2	2.2	1.1*
*Cytophagia*#	4.3	3.8	3.6	0.4	0.7	0.7
*Sphingobacteriia*#	2.0	1.5	1.5	0.5	0.2	0.1

*Firmicutes*#	0.2	0.4	0.3	9.9	6.1	5.6
*Bacilli*#	0.2	0.4	0.3*	9.9	6.0	5.5

*Chloroflexi*#	5.9	3.7	5.1	1.8	0.9	1.3
*Ktedonobacteria*	3.7	2.1	3.1	1.6	0.8	1.2

*Verrucomicrobia*#	1.8	1.9	1.3	0.1	0.1	0.1
*Acidobacteria*#	0.9	0.7	0.8	0.2	0.2	0.1
Other	16.1	9.8	11.2	13.4	12.7	12.0

# indicates a significant difference (*P*<0.05) between low-(LN) and high-nitrate (HN) conditions in the wild-type (Col-0).

* indicates a significant difference (*P*<0.05) between the wild-type and *nlp7-1* or *tcp20-4* according to Welch’s *t*-test. LN, no nitrate application; HN, high (240 mg N kg^−1^) nitrate supply. Values represent means (*n*=3).

**Table 3:** The relative abundance of 16S rRNA genes of major bacteria associated with roots of wild-type *Arabidopsis* at the family level.

Taxon	LN (%)	HN (%)	LN+HN (%)	P value	Fold change (HN/LN)
*Betaproteobacteria Oxalobacteraceae*	13.89	24.03	18.96	0.068	1.731
Unassigned	12.50	6.73	9.61	0.080	0.539
*Betaproteobacteria Burkholderiaceae*#	0.05	13.53	6.79	0.040	274
*Betaproteobacteria Comamonadaceae*#	11.69	1.02	6.36	0.015	0.088
*Actinobacteria Streptomycetaceae*	4.73	7.33	6.03	0.222	1.551
*Bacteroidetes Chitinophagaceae*#	7.49	3.16	5.33	0.003	0.422
*Betaproteobacteria Burkholderiales* Unassigned#	0.21	10.29	5.25	0.006	48.6
*Betaproteobacteria Rhodocyclaceae*	9.54	0.21	4.88	0.073	0.022
*Firmicutes Paenibacillaceae*#	0.09	9.50	4.80	0.022	103.615
*Gammaproteobacteria Xanthomonadaceae*	1.58	5.76	3.67	0.061	3.647
*Chloroflexi Ktedonobacteraceae*	3.66	1.57	2.61	0.064	0.429
*Cyanobacteria* SM1D11 Unassigned	0.24	4.59	2.41	0.053	19.118
*Deltaproteobacteria Myxococcales* Unassigned#	3.81	0.13	1.97	0.002	0.035
*Bacteroidetes Cytophagaceae*#	3.02	0.32	1.67	0.030	0.105
*Alphaproteobacteria Caulobacteraceae*#	1.50	0.82	1.16	0.010	0.545
*Gammaproteobacteria Legionellales* Unassigned	1.57	0.35	0.96	0.104	0.225
*Alphaproteobacteria Sphingomonadaceae*#	0.66	0.93	0.79	0.036	1.419
*Alphaproteobacteria Bradyrhizobiaceae*#	1.40	0.13	0.77	0.002	0.095
*Alphaproteobacteria Rhizobiaceae*#	1.46	0.06	0.76	0.013	0.043
*Verrucomicrobia Opitutaceae*#	1.33	0.03	0.68	0.026	0.021
*Bacteroidetes Sphingobacteriales* Unassigned#	1.28	0.06	0.67	0.050	0.044
*Actinobacteria Nocardiaceae*	0.42	0.88	0.65	0.251	2.119
*Bacteroidetes Cytophagales* Unassigned#	1.24	0.04	0.64	0.003	0.034
*Actinobacteria Actinomycetales* Unassigned	0.18	1.09	0.63	0.193	6.16
*Bacteroidetes Sphingobacteriaceae*	0.73	0.40	0.56	0.245	0.553
*Actinobacteria Micromonosporaceae*#	0.87	0.23	0.55	0.047	0.258
*Chloroflexi ouleothrixaceae*#	1.07	0.02	0.54	0.011	0.020
*Deltaproteobacteria Haliangiaceae*#	0.99	0.02	0.51	0.049	0.021

# indicates a significant difference (*P*<0.05) between low nitrate (LN) and high nitrate (HN) in the wild-type (Col-0) according to Welch’s *t*-test. LN, no nitrate application; HN, high (240 mg N kg^−1^) nitrate supply. Values represent means (*n*=3).

**Table 4:** The relative abundance of 16S rRNA genes of bacterial families associated with roots of wild-type *Arabidopsis* and *nlp7-1* and *tcp20-4* mutants.

Taxon	LN			HN		
	
	Col-0	*nlp7-1*	*tcp20-4*	Col-0	*nlp7-1*	*tcp20-4*
*Proteobacteria*						
*Sphingomonadaceae*#	0.66	0.89	1.23*	0.93	0.61*	0.56
*Hyphomicrobiaceae*#	0.31	0.24	0.16*	0.52	0.44	0.61
*Bacteriovoracaceae*#	0.15	0.07*	0.04*	0.01	0.00	0.00
*Myxococcaceae*#	0.06	0.01	0.03*	0.01	0.00	0.00
*Legionellales*_unclassified	1.57	0.54	1.32	0.35	0.16*	0.26
*Actinobacteria*						
*Actinomycetales*_unclassified	0.18	0.23	0.37*	1.09	0.42	0.71
*Nocardioidaceae*	0.17	0.23	0.40*	0.20	0.13	0.11*
*Micrococcaceae*#	0.04	0.06	0.06	0.16	0.06	0.08*
*Solirubrobacterales*_unclassified	0.02	0.06	0.01	0.04	0.01*	0.02
*Bacteroidetes*						
*Chitinophagaceae*#	7.49	7.39	8.35	3.16	2.24	1.14*
Others						
*Chthonomonadaceae*#	0.05	0.01*	0.04	0.01	0.00	0.00
*Parachlamydiaceae*#	0.23	0.05*	0.20	0.05	0.03	0.05
*Clostridiaceae*	0.03	0.01	0.01	0.01	0.04	0.04*

# indicates a significant difference (*P*<0.05) between low nitrate (LN) and high nitrate (HN) in the wild-type (Col-0).

* indicates a significant difference (*P*<0.05) between the wild-type and *nlp7-1* or *tcp20-4* according to Welch’s *t*-test. LN, no nitrate application; HN, high (240 mg N kg^−1^) nitrate supply. Values represent means (*n*=3).

**Table 5:** Comparison of bacterial functional gene frequencies in roots of wild-type *Arabidopsis* with those of *nlp7-1* and *tcp20-4* mutants under high-nitrate (HN) conditions

KEGG pathway	Gene frequency (HN)		

	Col-0	*nlp7-1*	*tcp20-4*
Metabolism			
Amino Acid Metabolism			
Lysine degradation	0.377	0.102*	0.291
Phenylalanine metabolism	0.299	0.081*	0.228
Tryptophan metabolism	0.495	0.132*	0.379
Valine, leucine, and isoleucine biosynthesis	0.475	0.130*	0.371
Valine, leucine, and isoleucine degradation	0.721	0.202*	0.559
beta-Alanine metabolism	0.379	0.103*	0.299
Cyanoamino acid metabolism	0.188	0.049*	0.139
D-Alanine metabolism	0.061	0.016*	0.045
Selenocompound metabolism	0.270	0.069*	0.205
Biosynthesis of Other Secondary Metabolites			
Butirosin and neomycin biosynthesis	0.031	0.009*	0.020
Penicillin and cephalosporin biosynthesis	0.093	0.022*	0.069
Phenylpropanoid biosynthesis	0.064	0.019*	0.038
Streptomycin biosynthesis	0.203	0.051*	0.147
Tropane, piperidine, and pyridine alkaloid biosynthesis	0.079	0.020*	0.060
Carbohydrate Metabolism			
Amino sugar and nucleotide sugar metabolism	0.746	0.194*	0.570
Ascorbate and aldarate metabolism	0.147	0.035*	0.103
Butanoate metabolism	0.826	0.228*	0.657
Citrate cycle (TCA cycle)	0.520	0.144*	0.409
Fructose and mannose metabolism	0.300	0.081*	0.231
Galactose metabolism	0.312	0.082*	0.222
Glycolysis/Gluconeogenesis	0.698	0.185*	0.539
Pentose and glucuronate interconversions	0.275	0.076*	0.199
Pentose phosphate pathway	0.480	0.125*	0.358
Propanoate metabolism	0.722	0.196*	0.557
Starch and sucrose metabolism	0.340	0.097*	0.232
Carbohydrate metabolism	0.058	0.018*	0.033
Lipid Metabolism			
alpha-Linolenic acid metabolism	0.036	0.009*	0.028
Biosynthesis of unsaturated fatty acids	0.193	0.053*	0.137
Ether lipid metabolism	0.057	0.013*	0.042
Fatty acid biosynthesis	0.417	0.109*	0.313
Fatty acid metabolism	0.606	0.163*	0.472
Glycerolipid metabolism	0.231	0.060*	0.171
Lipid biosynthesis proteins	0.664	0.173*	0.505
Sphingolipid metabolism	0.050	0.015*	0.029
Steroid hormone biosynthesis	0.018	0.005*	0.014
Synthesis and degradation of ketone bodies	0.145	0.039*	0.111
Metabolism of Terpenoids and Polyketides			
Biosynthesis of 12-, 14- and 16-membered macrolides	0.001	0.000*	0.000*
Carotenoid biosynthesis	0.022	0.005*	0.015
Geraniol degradation	0.249	0.072*	0.189
Limonene and pinene degradation	0.283	0.080*	0.214
Prenyltransferases	0.181	0.049*	0.133
Terpenoid backbone biosynthesis	0.310	0.085*	0.232
Xenobiotics Biodegradation and Metabolism			
Aminobenzoate degradation	0.392	0.106*	0.302
Benzoate degradation	0.462	0.129*	0.355
Bisphenol degradation	0.088	0.027*	0.064
Caprolactam degradation	0.168	0.048*	0.129
Chloroalkane and chloroalkene degradation	0.183	0.049*	0.138
Chlorocyclohexane and chlorobenzene degradation	0.051	0.018*	0.036
Dioxin degradation	0.055	0.014*	0.042
Drug metabolism - other enzymes	0.155	0.042*	0.121
Ethylbenzene degradation	0.064	0.019*	0.051
Fluorobenzoate degradation	0.036	0.010*	0.023
Naphthalene degradation	0.195	0.056*	0.152
Nitrotoluene degradation	0.054	0.018*	0.043
Polycyclic aromatic hydrocarbon degradation	0.083	0.027*	0.058
Toluene degradation	0.133	0.038*	0.096
Xylene degradation	0.022	0.007*	0.012
Other metabolic pathways			
Carbon fixation pathways in prokaryotes	0.692	0.188*	0.540
Methane metabolism	0.677	0.181*	0.530
Glycosyltransferases	0.245	0.063*	0.189
Retinol metabolism	0.061	0.017*	0.046
Thiamine metabolism	0.206	0.052*	0.150
Cellular Processes and Signaling			
Other transporters	0.160	0.039*	0.115
Environmental Information Processing			
Phosphotransferase system (PTS)	0.064	0.019*	0.050
Transporters	5.047	1.447*	4.043
Phosphatidylinositol signaling system	0.059	0.016*	0.046
Bacterial toxins	0.082	0.021*	0.062
Others			
Proteasome	0.031	0.008*	0.021
Protein export	0.317	0.082*	0.237
Base excision repair	0.337	0.086*	0.251
Mismatch repair	0.373	0.096*	0.280
Non-homologous end-joining	0.068	0.017*	0.048
Nucleotide excision repair	0.223	0.057*	0.165
Pathways in cancer	0.041	0.011*	0.032
Prostate cancer	0.020	0.005*	0.015
*Staphylococcus aureus* infection	0.001	0.000*	0.000
Tuberculosis	0.098	0.025*	0.073
Prion diseases	0.002	0.000*	0.001*
Adipocytokine signaling pathway	0.095	0.024*	0.073
PPAR signaling pathway	0.185	0.048*	0.141
Progesterone-mediated oocyte maturation	0.020	0.005*	0.015
Vasopressin-regulated water reabsorption	0.001	0.000*	0.000*
Antigen processing and presentation	0.020	0.005*	0.015
NOD-like receptor signaling pathway	0.020	0.005*	0.015

* indicates a significant difference (*P*<0.05) between the wild-type (Col-0) and *nlp7-1* or *tcp20-4* according to Welch’s *t*-test. HN, high (240 mg N kg^−1^) nitrate supply. Values represent means (*n*=3).
