# Supplementary material for: Nitrate Supply-Dependent Shifts in Communities of Root-Associated Bacteria in Arabidopsis
Source: Microbes Environ. 2017 Nov 30;32(4):314–23. doi: 10.1264/jsme2.ME17031 (PMC5745015; doi:10.1264/jsme2.ME17031)
Supplement: Supplementary file 1 [file 32_314_s1.pdf]

Supplementary materials

Nitrate supply–dependent shifts in communities of root-associated bacteria in *Arabidopsis*

Noriyuki Konishi[1,2,*], Takashi Okubo[3], Tomoyuki Yamaya[2], Toshihiki Hayakawa[1], and Kiwamu Minamisawa[4]

[1] Graduate School of Agricultural Science, Tohoku University, 468-1 Aramaki Aza Aoba, Sendai, Miyagi 980-0845, Japan

[2] Division for Interdisciplinary Advanced Research and Education, Tohoku University, 6-3 Aramaki Aza Aoba, Aoba-ku, Sendai, Miyagi 980-8578, Japan

[3] Institute for Agro-Environmental Sciences, National Agriculture and Food Research Organization, 3-1-3 Kannondai, Tsukuba, Ibaraki 305-8604, Japan

[4] Graduate School of Life Sciences, Tohoku University, Katahira, 2-1-1 Aoba-ku, Sendai, Miyagi 980-8577, Japan

Table S1. The number of sequences in each sample after filtering

		The number of sequences
LN	Col-0	12824
		22158
		12418
	nlp7-1	15054
		19687
		11805
	tcp20-4	9962
		15494
		14044
	Bulk soil	232288
		136638
		144056
HN	Col-0	8657
		9960
		4724
	nlp7-1	20569
		10008
		10315
	tcp20-4	9226
		9020
		5355
	Bulk soil	132773
		136311
		154429

LN, no nitrate application; HN, high (240 mg N kg^{-1}) nitrate application.

Table S2. The relative abundance of 16S rRNA genes of bacterial genera associated with the roots of wild-type *Arabidopsis* and *nlp7-1* and *tcp20-4* mutants

	LN			HN		
Taxon	Col-0	*nlp7-1*	*tcp20-4*	Col-0	*nlp7-1*	*tcp20-4*
Proteobacteria						
Comamonadaceae_unclassified	4.12	2.50*	3.40	0.30	0.09	0.04
Legionellales_unclassified	1.57	0.54	1.32*	0.35	0.16*	0.26
Kaistobacter	0.60	0.78	1.07*	0.91	0.59	0.54
Devosia	0.18	0.11	0.05*	0.08	0.14	0.22
Bacteriovoracaceae_unclassified	0.15	0.07*	0.04*	0.01	0.00	0.00
Rhodoplanes	0.13	0.11	0.08	0.40	0.28*	0.37
Rhodocyclaceae_unclassified	0.06	0.01*	0.00*	0.00	0.00	0.00
Sphingomonas	0.03	0.04	0.08*	0.01	0.01	0.01
Actinobacteria						
Actinoplanes	0.41	0.16*	0.49	0.07	0.00*	0.00*
Actinomycetales_unclassified	0.18	0.23	0.37*	1.09	0.42	0.71
Nocardioidaceae_unclassified	0.09	0.17	0.33*	0.16	0.11	0.08
Arthrobacter	0.04	0.06	0.06	0.15	0.05	0.07*
Solirubrobacterales_unclassified	0.02	0.06	0.01	0.04	0.01*	0.02
Bacteroidetes						
Chitinophagaceae_unclassified	6.45	6.29	7.20	3.03	2.21	1.12*
Other						
Parachlamydiaceae_unclassified	0.21	0.03*	0.18	0.04	0.03	0.05
DA101	0.08	0.03	0.03*	0.01	0.01	0.01
Chthonomonadaceae_unclassified	0.05	0.01*	0.04	0.01	0.00	0.00
Clostridium	0.01	0.01	0.01	0.01	0.02	0.04*
Edaphobacter	0.00	0.01	0.01	0.07	0.02	0.01*
Bacillaceae_unclassified	0.00	0.01	0.01	0.02	0.00*	0.02

LN, no nitrate application; HN, high (240 mg N kg^{-1}) nitrate application. * indicates significant difference ($P < 0.05$) between wild-type (Col-0) and *nlp7-1* or *tcp20-4*, respectively, according to Welch's *t*-test. Values represent mean (n=3).

Table S3. The relative abundance of 16S rRNA genes of bacterial OTUs associated with the roots of wild-type *Arabidopsis* and *nlp7-1* and *tcp20-4* mutants

	LN			HN					
OTU ID	Col-0	*nlp7-1*	*tcp20-4*	Col-0	*nlp7-1*	*tcp20-4*	Closest known species	Acc. No.	Identity (%)
Alphaproteobacteria									
573135	0.01	0.04	0.05*	0.02	0.00	0.00	*Bradyrhizobium ganzhouense*	NR_133706	98
849118	0.17	0.11	0.05*	0.00	0.00	0.01	*Devosia insulae*	NR_044036	99
207153	0.01	0.00	0.00	0.05	0.01*	0.02	*Azospirillum picis*	NR_042682	94
654742	0.03	0.00	0.08	0.57	0.40*	0.32	*Sphingomonas jaspsi*	NR_114034	97
Betaproteobacteria									
New.CleanUp.ReferenceOTU42690	0.06	0.01	0.01*	0.00	0.01	0.01	*Acidovorax delafieldii*	NR_116131	96
New.CleanUp.ReferenceOTU39462	0.01	0.00	0.00	0.04	0.06	0.20*	*Herbaspirillum massiliense*	NR_125602	96
1106324	0.14	0.01*	0.06	0.03	0.01	0.00	*Rhizobacter fulvus*	NR_041367	99
463546	0.06	0.01*	0.02	0.11	0.01	0.00	*Pelomonas saccharophila*	NR_114189	99
New.CleanUp.ReferenceOTU13204	0.03	0.00	0.00	0.35	0.57	1.12*	*Glaciimonas singularis*	NR_109670	96
546403	0.11	0.05	0.01*	0.15	0.46*	0.44	*Glaciimonas singularis*	NR_109670	98
941487	0.13	1.22	0.57*	0.00	0.00	0.02	*Massilia suwonensis*	NR_116872	99
849156	0.33	5.19	1.46*	0.06	0.01	0.01	*Massilia kyonggiensis*	NR_126273	99
4363537	1.36	1.54	0.40*	0.00	0.00	0.01	*Glaciimonas singularis*	NR_109670	98
574266	4.05	4.32	1.04*	0.00	0.00	0.01	*Glaciimonas singularis*	NR_109670	98
New.ReferenceOTU61	0.05	0.01*	0.00*	0.00	0.00	0.00	*Undibacterium seohonense*	NR_125672	94
New.ReferenceOTU235	0.17	0.04*	0.04*	0.21	0.01	0.01	*Uliginosibacterium gangwonense*	NR_043925	96
Deltaproteobacteria									
New.CleanUp.ReferenceOTU23776	0.11	0.06	0.03*	0.01	0.00	0.00	*Bacteriovorax stolpii*	NR_042023	96
1064689	0.05	0.06	0.11*	0.00	0.00	0.00	*Labilithrix luteola*	NR_126182	94
New.CleanUp.ReferenceOTU39703	0.01	0.01	0.05*	0.00	0.00	0.00	*Labilithrix luteola*	NR_126182	95
New.CleanUp.ReferenceOTU44337	0.23	0.11*	0.18	0.03	0.01	0.00	*Labilithrix luteola*	NR_126182	94
808919	0.13	0.18	0.06*	0.00	0.00	0.00	*Chondromyces lanuginosus*	NR_025345	97
New.CleanUp.ReferenceOTU36050	0.06	0.00*	0.04	0.01	0.00	0.00	*Chondromyces lanuginosus*	NR_025345	96
Gammaproteobacteria									
New.CleanUp.ReferenceOTU38253	0.04	0.00*	0.00*	0.01	0.00	0.00	*Rudaea cellulosilytica*	NR_044566	85
770614	0.04	0.01*	0.03	0.08	0.02*	0.01*	*Trabulsiella guamensis*	NR_114235	86
New.ReferenceOTU14	0.56	0.14	0.55	0.13	0.06	0.01*	*Ectothiorhodospira variabilis*	NR_042700	89
436590	0.05	0.02	0.01*	0.01	0.01	0.01	*Acidibacter ferrireducens*	NR_126260	95
Actinobacteria									
4366345	0.08	0.14	0.22*	0.01	0.01	0.00	*Catenulispora yoronensis*	NR_041613	99
104310	0.01	0.01	0.04*	0.01	0.01	0.00	*Jatrophihabitans soli*	NR_135866	96
4465540	0.00	0.00	0.00	0.04	0.00*	0.03	*Terrabacter koreensis*	NR_134212	99
554420	0.08	0.01*	0.06	0.04	0.04	0.00	*Catellatospora coxensis*	NR_041283	99
523680	0.40	0.16*	0.46	0.07	0.00*	0.00*	*Actinoplanes missouriensis*	NR_074576	98
996116	0.08	0.12	0.28*	0.01	0.00	0.00	*Nocardioides mesophilus*	NR_116027	99
1109208	0.00	0.01	0.00	0.04	0.01*	0.01	*Allokutzneria oryzae*	NR_133959	96
Firmicutes									
New.CleanUp.ReferenceOTU40122	0.06	0.01*	0.04	0.02	0.00	0.00	*Melghirimyces thermohalophilus*	NR_109725	87
606419	0.00	0.01	0.00	0.15	0.02*	0.12	*Bacillus luciferensis*	NR_025511	99
833317	0.04	0.09*	0.09	0.01	0.00	0.00	*Bacillus vireti*	NR_114096	99
251983	0.00	0.00	0.00	0.04	0.04	0.00*	*Paenibacillus frigoriresistens*	NR_109546	97
949660	0.01	0.02	0.00	6.68	1.85*	3.73	*Paenibacillus pectinilyticus*	NR_044487	98
New.CleanUp.ReferenceOTU12634	0.00	0.01	0.01	0.37	0.05*	0.27	*Melghirimyces thermohalophilus*	NR_109725	87
Others									
568398	0.00	0.01	0.00	0.07	0.01*	0.01*	*Edaphobacter modestus*	NR_115813	98

353494	0.91	0.66*	1.18	1.18	0.88	0.40	*Niastella koreensis*	NR_074595	99
New.CleanUp.ReferenceOTU44361	0.01	0.03	0.06*	0.01	0.00	0.00	*Thermomarinilinea lacunifontana*	NR_132293	87
217917	0.11	0.01*	0.08	0.01	0.00	0.00	*Oscillochloris trichoides*	NR_114470	81
New.ReferenceOTU283	3.28	1.80	2.70	0.08	0.04*	0.02*	*Ktedonobacter racemifer*	NR_112949	88
New.ReferenceOTU28	0.42	0.13*	0.32	0.07	0.04	0.23	*Cephalothrix komarekiana CCIBt*	NR_137273	79
248401	0.05	0.04	0.01*	0.00	0.00	0.00	*Limisphaera ngatamarikiensis*	NR_134756	92
New.ReferenceOTU12	3.44	1.33	1.23*	0.01	0.01	0.00	Unknown		
New.CleanUp.ReferenceOTU20281	0.04	0.03	0.01*	0.01	0.00	0.00	Unknown		

LN, no nitrate application; HN, high (240 mg N kg^{-1}) nitrate application. * indicates significant difference ($P < 0.05$) between wild-type (Col-0) and *nlp7-1* or *tcp20-4*, respectively, according to Welch's *t*-test. Values represent mean (n=3).

Table S4. Comparison of bacterial functional gene frequency in the roots of wild-type *Arabidopsis* with those of *nlp7-1* and *tcp20-4* mutants in the low-nitrate (LN) condition

KEGG pathway	Gene frequency (LN)		
	Col-0	*nlp7-1*	*tcp20-4*
Biosynthesis of Other Secondary Metabolites			
Caffeine metabolism	0.001	0.002	0.003*
Clavulanic acid biosynthesis	0.001	0.001	0.003*
Flavone and flavonol biosynthesis	0.007	0.004*	0.007
Indole alkaloid biosynthesis	0.004	0.008	0.006*
Isoflavonoid biosynthesis	0.000	0.001	0.001*
Glycan Biosynthesis and Metabolism			
Glycosaminoglycan degradation	0.045	0.029*	0.051
Glycosphingolipid biosynthesis - ganglio series	0.033	0.021*	0.038
Glycosphingolipid biosynthesis - lacto and neolacto series	0.001	0.005	0.003*
Other glycan degradation	0.137	0.088*	0.159
Glycan biosynthesis and metabolism	0.149	0.235	0.079*
Lipid Metabolism			
Secondary bile acid biosynthesis	0.008	0.008	0.002*
Synthesis and degradation of ketone bodies	0.366	0.479	0.245*
Lipid metabolism	0.445	0.679	0.274*
Metabolism of Other Amino Acids			
beta-Alanine metabolism	0.892	1.163	0.623*
Glutathione metabolism	1.013	1.460	0.624*
Phosphonate and phosphinate metabolism	0.171	0.248	0.102*
Metabolism of Terpenoids and Polyketides			
Biosynthesis of 12-, 14- and 16-membered macrolides	0.005	0.002*	0.005
Biosynthesis of siderophore group nonribosomal peptides	0.090	0.131	0.063*
Biosynthesis of type II polyketide products	0.000	0.001	0.002*
Limonene and pinene degradation	0.776	1.000	0.539*
Xenobiotics Biodegradation and Metabolism			
1,1,1-Trichloro-2,2-bis(4-chlorophenyl)ethane (DDT) degradation	0.005	0.003*	0.006
Aminobenzoate degradation	1.016	1.408	0.676*
Benzoate degradation	1.378	1.806	0.869*
Chlorocyclohexane and chlorobenzene degradation	0.297	0.371	0.157*
Dioxin degradation	0.141	0.169	0.095*
Drug metabolism - cytochrome P450	0.499	0.713	0.311*
Ethylbenzene degradation	0.144	0.198	0.098*
Fluorobenzoate degradation	0.212	0.280	0.112*
Metabolism of xenobiotics by cytochrome P450	0.483	0.702	0.290*
other Metabolisms			
Glyoxylate and dicarboxylate metabolism	1.746	2.376	1.080*
Protein kinases	0.878	1.300	0.603*
Cellular Processes and Signaling			
Apoptosis	0.016	0.016	0.009*
p53 signaling pathway	0.005	0.002*	0.004
Bacterial motility proteins	4.109	6.775	2.499*
Lysosome	0.086	0.054*	0.096
Cell division	0.169	0.247	0.100*
Cell motility and secretion	0.614	0.935	0.352*

	Electron transfer carriers	0.011	0.008	0.015*
	Sporulation	0.020	0.029*	0.033
Environmental Information Processing				
	ABC transporters	7.935	10.989	4.839*
	Transporters	12.104	16.888	7.639*
	Two-component system	4.854	7.412	3.081*
	Cellular antigens	0.185	0.242	0.114*
	Ion channels	0.011	0.033	0.020*
Others				
	Cardiac muscle contraction	0.118	0.180	0.067*
	Bile secretion	0.001	0.001	0.002*
	RIG-I-like receptor signaling pathway	0.001	0.001	0.003*
	Ubiquitin system	0.015	0.009*	0.019
	Transcription factors	2.775	3.996	1.831*
	Colorectal cancer	0.005	0.002*	0.004
	Small cell lung cancer	0.005	0.002*	0.004
	Viral myocarditis	0.005	0.002*	0.004
	Epithelial cell signaling in Helicobacter pylori infection	0.058	0.098	0.038*
	Influenza A	0.005	0.002*	0.004
	Pertussis	0.255	0.380	0.128*
	Staphylococcus aureus infection	0.006	0.004*	0.007
	Toxoplasmosis	0.005	0.002*	0.004
	Huntington's disease	0.188	0.261	0.111*
	Parkinson's disease	0.124	0.184	0.072*
	Prion diseases	0.011	0.010	0.004*

LN, no nitrate application. * indicates significant difference ($P < 0.05$) between wild-type and *nlp7-1* or *tcp20-4*, respectively, according to Welch's *t*-test. Values represent mean (n=3).

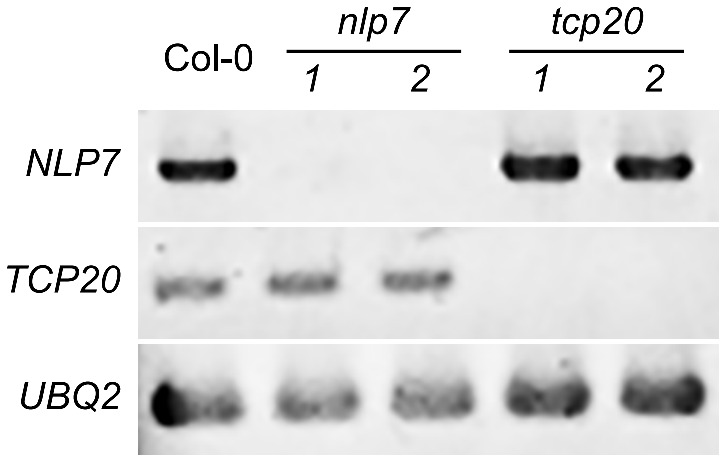

Fig. S1. Isolation of single-insertion lines for *NLP7* or *TCP20* gene. Reverse transcription polymerase chain reaction analysis of RNA from whole plants of *NLP7* and *TCP20* insertion lines. Plants were cultured on half-strength Murashige and Skoog medium agar plates for 25 days.

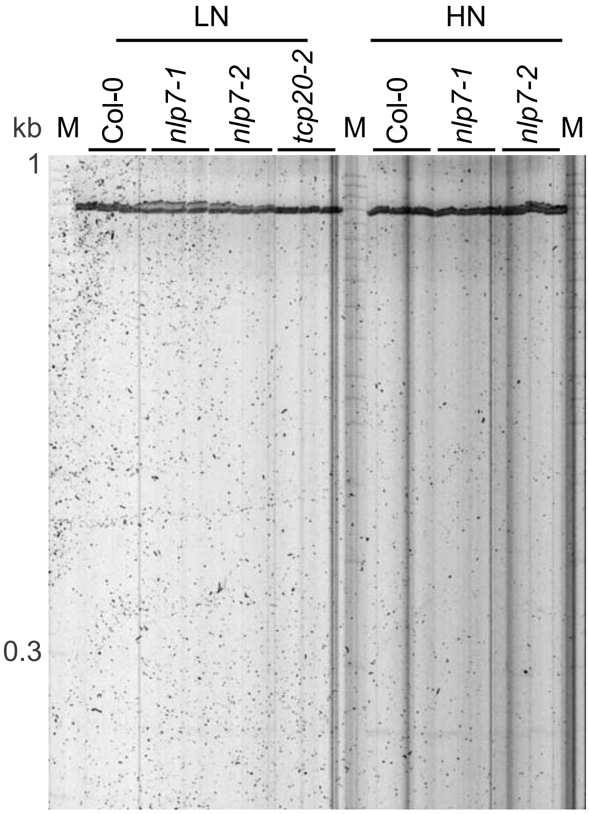

Fig. S2. RISA profiles of root-associated fungal communities in wild-type *Arabidopsis* (Col-0) and *nlp7-1*, *nlp7-2*, and *tcp20-2* mutants. LN, no nitrate application; HN, high (240 mg N kg^{-1}) nitrate application.
